# Serum lithium test requesting across three UK regions: an evaluation of adherence to monitoring guidelines

**DOI:** 10.1186/s12888-020-03023-y

**Published:** 2021-01-12

**Authors:** Ceri Parfitt, Christopher J. Duff, Jonathan Scargill, Lewis Green, David Holland, Adrian H. Heald, Anthony A. Fryer

**Affiliations:** 1 Department of Clinical Biochemistry, University Hospitals of North Midlands NHS Trust, Stoke-on-Trent, Staffordshire UK; 2 School of Medicine, Keele University, Keele, Stoke-on-Trent, Staffordshire UK; 3 Department of Clinical Biochemistry, The Royal Oldham Hospital, The Northern Care Alliance NHS Group, Oldham, UK; 4 St. Helens & Knowsley Teaching Hospitals NHS Trust, Whiston Hospital, Prescot, UK; 5 The Benchmarking Partnership, Alsager, Cheshire UK; 6 Salford Royal Hospital NHS Foundation Trust, The Northern Care Alliance NHS Group, Salford, UK; 7 The School of Medicine and Manchester Academic Health Sciences Centre, University of Manchester, Manchester, UK; 8 Professor of Clinical Biochemistry, University Hospitals of North Midlands NHS Trust, Keele University, Stoke-on-Trent, Staffordshire ST5 5BG UK

**Keywords:** Lithium, Bipolar disorder, Monitoring, Guidelines, Serum lithium concentration, Testing frequency, Inappropriate test utilisation

## Abstract

**Background:**

Bipolar disorder is the fourth most common mental health condition, affecting ~ 1% of UK adults. Lithium is an effective treatment for prevention of relapse and hospital admission, and is widely recommended as a first-line treatment. We previously showed in other areas that laboratory testing patterns are variable with sub-optimal conformity to guidance. We therefore examined lithium results and requesting patterns relative to monitoring recommendations.

**Methods:**

Data on serum lithium levels and intervals between requests were extracted from Clinical Biochemistry laboratory information systems at the University Hospitals of North Midlands, Salford Royal Foundation Trust and Pennine Acute Hospitals from 2012 to 2018 (46,555 requests; 3371 individuals). Data were examined with respect to region/source of request, age and sex.

**Results:**

Across all sites, lithium levels on many requests were outside the recommended UK therapeutic range (0.4–0.99 mmol/L); 19.2% below the range and 6.1% above the range (median [Li]: 0.60 mmol/L). A small percentage were found at the extremes (3.2% at < 0.1 mmol/L, 1.0% at ≥1.4 mmol/L). Most requests were from general practice (56.3%) or mental health units (34.4%), though those in the toxic range (≥1.4 mmol/L) were more likely to be from secondary care (63.9%). For requesting intervals, there was a distinct peak at 12 weeks, consistent with guidance for those stabilised on lithium therapy. There was no peak at 6 months, as recommended for those aged < 65 years on unchanging therapy, though re-test intervals in this age group were more likely to be longer. There was a peak at 0–7 days, reflecting those requiring closer monitoring (e.g. treatment initiation, toxicity). However, for those with initial lithium concentrations within the BNF range (0.4–0.99 mmol/L), 69.4% of tests were requested outside expected testing frequencies.

**Conclusions:**

Our data showed: (a) lithium levels are often maintained at the lower end of the recommended therapeutic range, (b) patterns of lithium results and testing frequency were comparable across three UK sites with differing models of care and, (c) re-test intervals demonstrate a noticeable peak at the recommended 3-monthly, but not at 6-monthly intervals. Many tests were repeated outside expected frequencies, indicating the need for measures to minimise inappropriate testing.

## Background

Bipolar disorder is the 4th most common mental health condition, affecting approximately 1% of adults [1]. Individuals with bipolar disorder typically have recurrent episodes of elevated mood (mania) and periods of depressed mood, which may last for several weeks. A combination of therapies is often required to manage different aspects of bipolar disorder, including pharmacological treatments, psychological therapies and lifestyle advice.

Lithium is the most effective treatment for prevention of relapse and hospital admission in people with bipolar disorder, and is recommended by The National Institute for Health and Care Excellence (NICE) in the UK as a first line long-term treatment [2], as well as in clinical practice guidelines in the USA, Canada, Japan, the Netherlands, and Australia and New Zealand, and in the International Society for Bipolar Disorders [3–5]. Lithium is also used to treat other conditions such as recurrent depression [6].

However, lithium treatment is associated with both short-term and long-term risks. Insufficient dose, poor adherence or sudden discontinuation of lithium can result in relapse. In contrast, acute lithium toxicity can presents with a variety of clinical manifestations including renal, neurological, gastrointestinal, cardiac and endocrine abnormalities [7].

Because of these factors, maintaining blood lithium concentration within a relatively narrow therapeutic index is desirable. NICE guidelines currently advise maintaining serum lithium concentration between 0.6 and 0.8 mmol/L, or between 0.8 and 1.0 mmol/L in people who have relapsed whilst taking lithium, or people who have sub-threshold symptoms with functional impairment [2]. The British National Formulary (BNF) recommends that serum lithium is maintained within the range 0.4–1.0 mmol/L, focusing on the lower end of this range for those on maintenance therapy and in elderly patients [6].

Recommended monitoring intervals for lithium vary according to individual status. For people initiating lithium therapy, NICE guidelines recommend weekly monitoring until a stable baseline is established [2]. Subsequently, it is suggested that serum lithium be monitored on a three-monthly basis for the first year of treatment, increasing to six-monthly for people under 65 years of age with no changes affecting lithium concentration. More frequent monitoring may be initiated for a variety of reasons, including dose and formulation changes, changing other medications or intercurrent illness. In particular, individuals with potentially toxic serum lithium concentrations (> 1.4 mmol/L) should have serial daily lithium measurements taken to ensure elimination and avoid rebound toxicity [8].

We have shown in other areas that laboratory testing patterns are highly variable and that conformity to guidance in sub-optimal [9–11]. This study therefore aims to assess lithium results and patterns of requesting, and compare these findings to current guidance on lithium requesting. We examined these using clinical laboratory data collected from three large UK centres, where the approach to managing patients with bipolar disorder and ordering lithium testing varies.

## Methods

### Data collection

All lithium requests received by the Clinical Biochemistry Departments at the University Hospitals of North Midlands (UHNM), Salford Royal Foundation Trust (SRFT) and Pennine Acute Hospitals NHS Trust (PAT) between 2012 and 2018 were extracted from the respective Laboratory Information and Management Systems (49,584 requests). These sites represented mixed rural and urban populations. Each used different approaches to monitoring lithium therapy in terms of the relative proportion of tests requested by primary and specialist care. People with a single lithium request, those initiating lithium therapy in the final year of data collection and those under the age of 18 were excluded, leaving a data set of 46,555 requests from 3371 individuals.

### Data categorisation

Sources of request were categorised as General Practices (GPs), Mental Health Units (MHUs; including inpatient and outpatient requests), Acute Care (including Emergency Departments, acute medical & surgical units, etc.), Secondary Care (all acute hospital inpatient and outpatient, excluding Acute Care sources) and Other (including unknown sources). The demographics of this data set are shown in Table 1.

**Table 1:** Study demographics

		PAT	%	SRFT	%	UHNM	%	Total	%
**Number of patients**	**Total**	1613		1162		596		3371	
**Sex**	**Male**	670	41.5	469	40.4	238	39.9	1377	40.8
	**Female**	943	58.5	690	59.4	358	60.1	1991	59.1
**Age (years)**	**Median (IQR)**	50 (39–62)		53 (41–68)		54 (42–67)		52 (40–65)	
**Number of requests**	**GP**	17,118	73.4	5890	44.7	3215	32.0	26,223	56.3
	**MHU**	3938	16.9	6101	46.3	5996	59.6	16,035	34.4
	**Secondary Care**	1658	7.1	642	4.9	559	5.6	2859	6.1
	**Acute Care**	461	2.0	470	3.6	177	1.8	1108	2.4
	**Other**	144	0.6	71	0.5	115	1.1	330	0.7
	**Total**	23,319	50.1	13,174	28.3	10,062	21.6	46,555	
**Requests per patient**		14.5		11.3		16.9		13.8	

Abbreviations and definitions: *PAT* Pennine Acute Trust, *SRFT* Salford Royal Foundation Trust, *UHNM* University Hospitals of North Midlands, *GP* General Practice, *MHU* Mental Health Unit (including inpatient and outpatient), Secondary Care: Acute hospital inpatient and outpatient, excluding Acute Care sources; Acute Care: Acute hospital Emergency Departments and acute medical/surgical units; Other: all other sources of requests, including unknown sources, *IQR* Inter-quartile range

Lithium concentrations were grouped into categories: < 0.10 mmol/L (undetectable by the laboratory assay); 0.10–0.39 mmol/L (below the BNF recommended therapeutic range and where lithium therapy is generally ineffective) [6, 12]; 0.4–0.59 mmol/L (within the BNF range, but below the NICE therapeutic range) [2, 6]; 0.60–0.79 mmol/L (within both BNF and NICE ranges); 0.8–0.99 mmol/L (within both BNF and NICE ranges, but where BNF recommends more frequent monitoring) [6]; 1.0–1.39 mmol/L (above both BNF and NICE ranges, and where very few patients gain additional benefit) [2, 6, 12] and ≥1.4 mmol/L (were toxicity is likely) [13].

Intervals between lithium tests were calculated as number of days until the next lithium result was requested for each person.

### Data analysis

This study represented a service evaluation and audit of practice against the guidelines outlined by NICE and the BNF [2, 6]. According to the decision tool provided by the UK Health Research Authority [14], this study was not considered to be research and did not require NHS Research Ethics Committee review. It therefore did not require ethical committee approval. All data extracted from Laboratory Information and Management Systems were anonymised and local Trust processes were used to obtain approval for use of the data.

All statistical analyses were performed using Stata (version 14; College Station, TX). We used the Kruskal-Wallis test for comparisons of median lithium concentrations across sites and Mann-Whitney U test for comparisons between males and females. Linear regression was used to assess the association between lithium concentration with age. Chi-squared analyses were used to compare differences in proportions within categories (lithium concentration or requesting interval groups) between sites, source of request, gender and age groups.

## Results

### Demographics

Table 1 shows a demographic summary of people included in the study. At all three trusts, the majority of requests came from either GP practices or MHUs, with a minority from acute care units, secondary care, or other sources. However, the proportion of requests from GP practices compared to MHUs varied between Trusts. At PAT, GP requests comprised 73.4% of total requests, whereas at UHNM, most requests originated from MHU (59.6%; GP practice requests comprised 32.0%). At SRFT, GP and MHU requests were evenly split (44.7% and 46.3%, respectively).

### Lithium concentrations

Overall, the median lithium concentrations were at the lower limit of the therapeutic range (0.60 mmol/L; IQR 0.44–0.76) (Table 2). The median lithium concentrations were generally lowest in samples from the SRFT and highest from UHNM (*p*< 0.001, Kruskal-Wallis test) and were slightly higher in females (0.60 mmol/L; IQR 0.45–0.76) than males (0.59 mmol/L; IQR 0.43–0.75; *p<* 0.001, Mann-Whitney U test). There was also a statistically significant positive correlation between lithium concentration and age (*p<* 0.001, linear regression), though the strength of this association was not clinically meaningful (*r*=0.07).

**Table 2:** Detailed breakdown of serum lithium concentration profile by site and source of requests

	Lithium concentration (mmol/L)								Median (IQR) lithium concentration (mmol/L)
	< 0.1	0.1–0.39	0.4–0.59	0.6–0.79	0.8–0.99	1.0–1.39	≥1.4	Total	
**Site**									
**PAT**	3.5%	16.0%	**29.9%**	**30.2%**	**14.3%**	5.1%	1.1%	100.0%	0.60 (0.44–0.76)
**SRFT**	3.3%	17.5%	**32.6%**	**29.9%**	**12.0%**	3.9%	0.8%	100.0%	0.58 (0.43–0.73)
**UHNM**	2.6%	14.0%	**27.4%**	**30.5%**	**17.9%**	6.4%	1.2%	100.0%	0.63 (0.47–0.80)
**Total**	3.2%	16.0%	**30.1%**	**30.2%**	**14.4%**	5.1%	1.0%	100.0%	0.60 (0.44–0.76)
**Source**									
**GP**	53.7%	47.7%	**57.2%**	**60.0%**	**60.6%**	53.7%	24.4%	56.4%	0.61 (0.46–0.76)
**MHU**	31.3%	39.1%	**35.6%**	**33.9%**	**32.4%**	28.3%	11.1%	34.4%	0.58 (0.42–0.74)
**Acute care**	4.5%	3.0%	**1.5%**	**1.1%**	**1.8%**	6.0%	38.0%	2.4%	0.66 (0.38–1.10)
**Secondary care**	9.3%	9.2%	**5.0%**	**4.3%**	**4.7%**	11.3%	25.9%	6.1%	0.53 (0.34–0.75)
**Other**	1.3%	1.0%	**0.6%**	**0.7%**	**0.5%**	0.7%	0.6%	0.7%	0.57 (0.38–0.68)
**Total**	100.0%	100.0%	100.0%	100.0%	100.0%	100.0%	100.0%	100.0%	

The categories within the NICE- and BNF-recommended therapeutic range are highlighted in bold: 0.4–0.59 mmol/L for BNF extension to the NICE-recommended range, 0.6–0.79 mmol/L for NICE-recommended range for people receiving routine lithium treatment; 0.8–0.99 mmol/L for NICE-recommended range for people who have relapsed whilst taking lithium, or people who have sub-threshold symptoms with functional impairment

The proportion of lithium tests in the key categories referred to in guidance showed that, in the overall dataset, 74.7% of results were within the 0.4–0.99 mmol/L range, with the majority of these in the lower part of this range. Approximately 30% of lithium results fell into 0.4–0.59 mmol/L range (within the BNF recommended range).

**Fig. 1:**
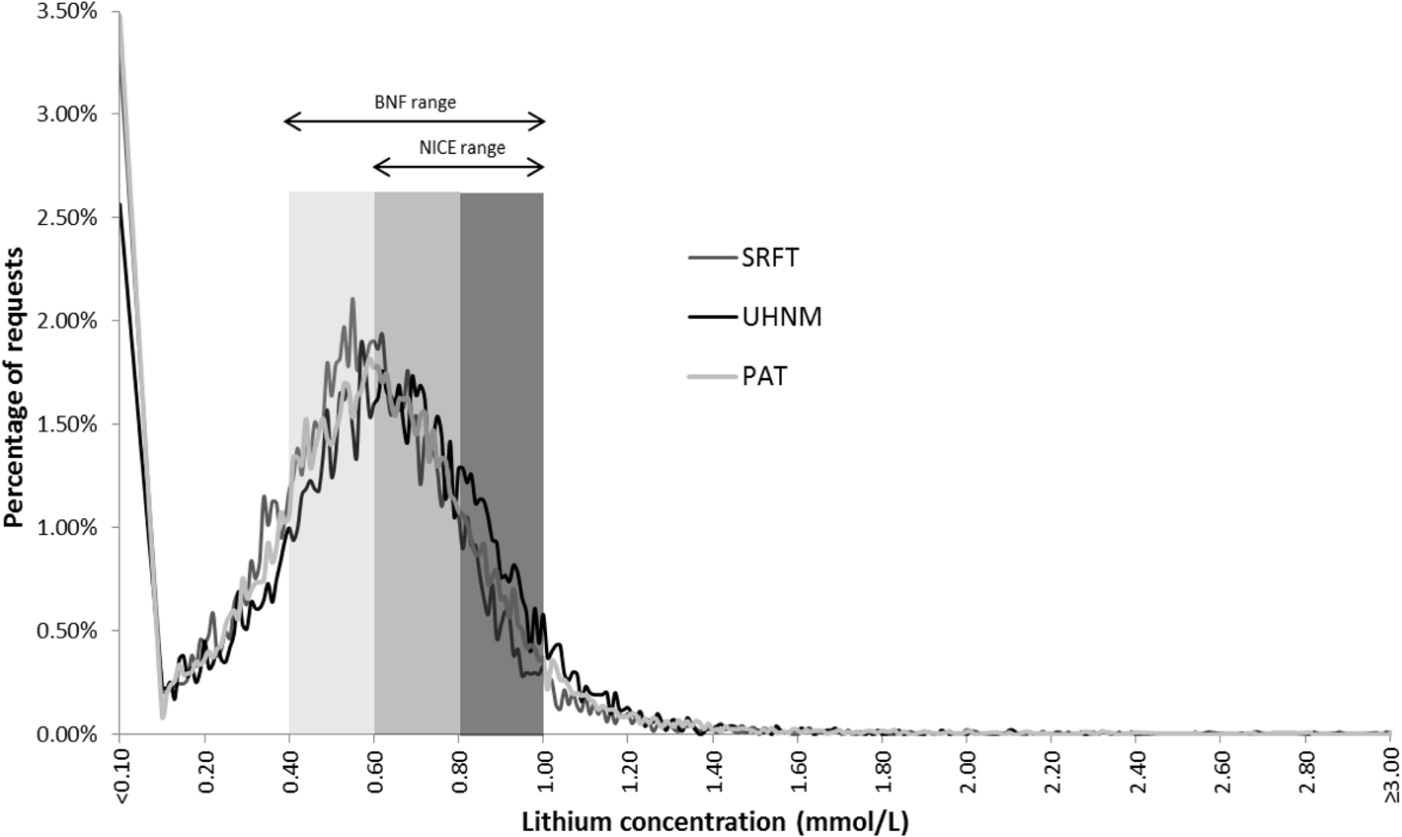
Serum lithium concentration profile for each site. The categories within the NICE- and BNF-recommended therapeutic range is indicated by shading: light grey for BNF extension to the NICE-recommended range (0.4–0.59 mmol/L), mid grey for NICE-recommended range for people receiving routine lithium treatment (0.6–0.79 mmol/L); dark grey for NICE-recommended range for people who have relapsed whilst taking lithium, or people who have sub-threshold symptoms with functional impairment (0.8–0.99 mmol/L)

The distribution of lithium concentrations from requests across the three Trusts (Fig. 1) showed that the lithium concentration profile for each of the Trusts were broadly similar, with a peak at approximately 0.6 mmol/L. This indicated that almost half of results (49.3%) were below the NICE recommended therapeutic window, while only 6% were above the window. A large peak was noted at < 0.1 mmol/L; reflecting results below the detectable range of the assay. When examined in terms of proportions within the lithium concentration categories between sites (Table 2), while the overall patterns were broadly similar, they were statistically different (χ^2^_12_=332.5, *p*< 0.001) with a slightly higher proportion of results from UHNM within the range 0.8–0.99 mmol/L and lower proportion in the < 0.1 and 0.1–0.39 mmol/L categories. This was reflected in the higher overall median lithium concentration for UHNM. A small percentage of results were found at the extremes - less than 4% at < 0.1 mmol/L and less than 2% at > 1.4 mmol/L – across all three sites.

Similarly, lithium requests falling into each category were split by source (Table 2). This demonstrated that the distribution of source of request differed significantly by lithium concentration (χ^2^_24_=4002, *p*< 0.001). For those cases were tests were within the range 0.4–0.99 mmol/L, most tests were requested by GPs or MHUs, supporting the view that most routine management of lithium dosing for those on stable therapy is being performed by these units. For results in the two toxic ranges (1.0–1.39 and ≥1.4 mmol/L), a greater proportion were requested from acute and, to a lesser extent, secondary care when compared with non-toxic levels. This was reflected in the higher median lithium concentration for requests from acute care sources.

### Requesting intervals

**Fig. 2:**
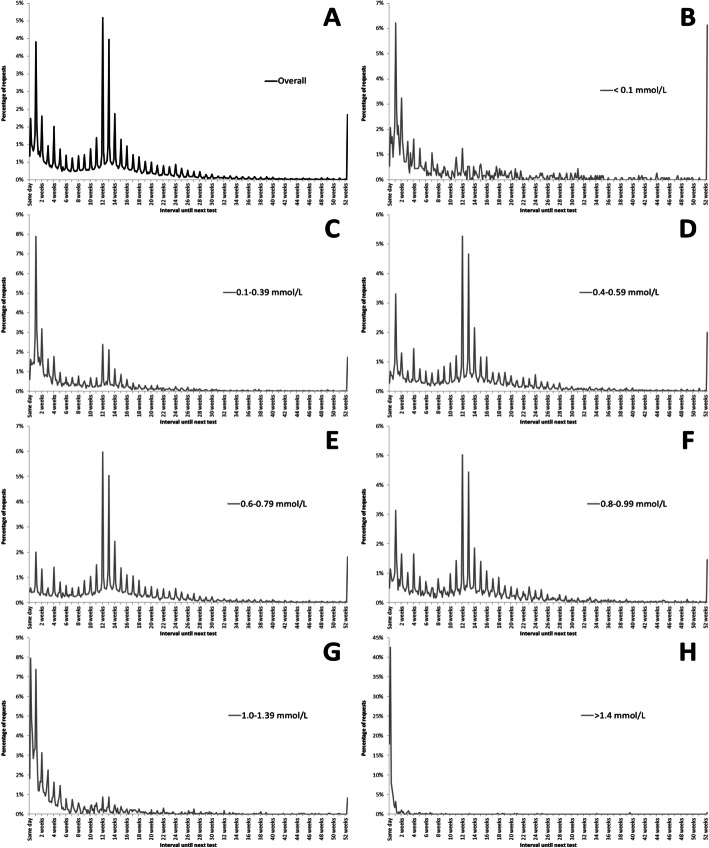
Frequency of intervals between consecutive lithium requests (truncated at 1 year). Percentages of requests reflect daily requests and are show for the total number of requests (panel **a**) and categorised by initial lithium concentration (panels **b-h**)

Figure 2 shows the relative frequencies of intervals between pairs of requests for the total group (Fig. 2a) and for each category of lithium concentration (Fig. 2b-h). Overall, there was a distinct peak at 12 weeks, as suggested in NICE and BNF guidance for those stabilised on lithium therapy. There was no peak evident at 6 months as suggested in NICE guidance for those less than 65 years old on unchanging therapy. As 22,732 requests were from the 15,514 people aged < 65 years of age whose initial lithium concentration was in the range 0.4–0.99 mmol/L, we would have expected a distinct peak of test requests at 6 months. Moving these cases from 3- to 6-monthly testing would reduce the number of lithium request by up to 6644 per year across the regions serviced by these three laboratories.

There was a peak at 0–7 days, reflecting those requiring closer monitoring (e.g. at treatment initiation or with results in the toxic range). It was also noted that there were spikes of tests requested at weekly intervals throughout, suggesting that there may be a weekly recurring clinic at which samples were collected. There were no noticeable differences in overall pattern when stratified by site (data not shown).

We also examined the pattern of requesting intervals based on initial lithium result. This showed distinct patterns of request interval for each category of lithium result (Fig. 2b-h). For results within the NICE and BNF recommended range categories (0.4–0.59 mmol/L, 0.6–0.79 mmol/L and 0.8–0.99 mmol/L), the modal interval was 84 days (12 weeks). In contrast, peaks are noted at much earlier intervals for other categories: at 7 days for < 0.1 mmol/L and 0.1–0.39 mmol/L concentrations; and at 1 day for 1.0–1.39 mmol/L and > 1.4 mmol/L concentrations.

Tables 3 and 4 shows the proportion of cases within defined interval categories, split by sex, age (Table 3) and initial lithium concentration (Table 4). While broadly similar, the pattern of test requesting intervals differed statistically by both sex (χ^2^_7_=106.4, *p*< 0.001) and age (χ^2^_7_=370.4, *p<* 0.001). Hence, intervals were more likely to be shorter in females (46.3% less than 11 weeks) compared with males (43.0% less than 11 weeks; *p<* 0.001, OR 1.15, 95% CI 1.10–1.19). Those patients aged over 65 years (66.7% less than 14 weeks) were also more likely to have shorter intervals compared with those aged ≤65 years (58.5% less than 14 weeks; *p<* 0.001, OR 1.42, 95% CI 1.36–1.48).

**Table 3:** Intervals between lithium requests stratified by sex, and age

Interval between requests	Sex		Age (years)	
	Male	Female	≤65	> 65
**0–1 days**	2.3%	2.6%	2.1%	3.4%
**2–7 days**	7.9%	9.7%	8.4%	10.3%
**8–76 days (weeks 2 to 10)**	32.8%	34.1%	32.7%	35.4%
**77–98 days (weeks 11 to 13)**	16.3%	16.0%	15.4%	17.6%
**99–160 days (weeks 14 to 22)**	25.1%	24.8%	26.0%	22.5%
**161–189 days (weeks 23 to 26)**	3.0%	2.7%	3.0%	2.4%
**190–365 (weeks 27 to 51)**	10.5%	8.5%	10.3%	7.2%
**> 365 (weeks 52 and above)**	2.1%	1.7%	2.2%	1.1%
**Total**	**100.0%**	**100.0%**	**100.0%**	**100.0%**

**Table 4:** Intervals between lithium requests stratified by initial lithium concentrations

Interval between requests	Lithium concentration (mmol/L)^**a**^						
	< 0.1	0.1–0.39	0.4–0.59	0.6–0.79	0.8–0.99	1.0–1.39	> 1.4
**0–1 days**	2.6%	2.2%	1.0%	1.0%	1.6%	**9.8%**	**60.5%**
**2–7 days**	**14.1%**	**16.2%**	6.5%	4.3%	7.4%	**25.6%**	26.6%
**8–76 days (weeks 2 to 10)**	**45.6%**	**46.4%**	30.4%	27.8%	33.0%	46.0%	9.9%
**77–98 days (weeks 11 to 13)**	7.6%	13.1%	**24.5%**	**27.0%**	**23.0%**	6.1%	0.2%
**99–160 days (weeks 14 to 22)**	11.7%	12.6%	22.0%	23.7%	21.1%	7.3%	1.3%
**161–189 days (weeks 23 to 26)**	3.4%	2.7%	**5.2%**	**5.9%**	**4.9%**	1.5%	0.4%
**190–365 (weeks 27 to 51)**	8.8%	5.0%	8.5%	8.6%	7.5%	2.9%	0.6%
**> 365 (weeks 52 and above)**	6.1%	1.7%	2.0%	1.8%	1.5%	0.8%	0.4%
**Total**	**100.0%**	**100.0%**	**100.0%**	**100.0%**	**100.0%**	**100.0%**	**100.0%**

Categories where the majority of tests would be expected based on guidelines are highlighted in bold

^a^lithium concentration was based on the result of the first lithium of each pair of tests that define an interval

Table 4 shows the link between testing interval and lithium concentration and illustrates the large proportion of tests requested outside recommended retesting intervals. For example, for those with initial lithium concentrations within the range 0.4–0.99 mmol/L where the recommended intervals (with the exceptions defined in the Introduction) is generally 12 weeks or 26 weeks, a large proportion were requested either before this time (< 11 weeks; 36.7%), in the gap between these two recommended intervals (weeks 14–22; 22.5%) or later than recommended > 27 weeks (10.2%). In those cases in the toxic range > 1 mmol/L, there were a number that were requested later than recommended: 64.6% were re-tested later than 7 days in those with an initial lithium level of 1.0–1.39 mmol/L and 39.5% were retested later than 1 day in those with initial lithium concentrations of > 1.4 mmol/L. Similarly, those in the < 0.4 mmol/L category might be expected to be re-checked at 1–2 weeks during lithium dose up-titration, for example. However, 40.3% were re-tested outside the 2–7 days and 8–76 days categories.

## Discussion

The use of lithium as a treatment for bipolar disorder has been established for over forty years. In the UK, USA, Canada, Japan, the Netherlands and Australia and New Zealand, lithium is currently recommended as a first line treatment for bipolar disorder [1–5]. Lithium has a low therapeutic index, with a narrow interval between therapeutic and toxic doses. People taking lithium therefore need to ensure they receive sufficient dosage for clinical effect, whilst minimising the risk of side effects and toxicity. If tolerated, lithium has been shown to be an effective treatment for bipolar disorder. Improper dosing may lead to non-adherence, prescription of additional or alternative medication, or failure of therapy, leading to relapse.

### Lithium levels

In our study, the mean serum lithium concentration was found to be around 0.6 mmol/L across all three centres. This is at the lower end of the NICE recommended range [2], but within that recommended by the BNF [6]. Indeed, the overall pattern of lithium concentrations was very similar across the three centres suggesting that, despite differences in proportion of tests requested by general practices and MHUs, there is consensus on target levels. Approximately 30% of results were between 0.6 and 0.8 mmol/L and a further 30% between 0.4 and 0.6 mmol/L. The finding that around 45% of results fall into the range recommended by NICE for the majority of our patient population (0.6–1.0 mmol/L) is in keeping with the findings of Nikolova et al. [4] who found that serum levels were within this range in 50.7% of cases.

Although it may appear concerning that such a large proportion of lithium test results are outside the NICE recommended therapeutic range, this may be indicative of widespread use of the BNF ranges in local guidelines, or pragmatic prescribing by clinicians or inconsistencies between individual recommendations, as summarised by Nederlof et al. [3]. Local Shared Care Agreements covering the three centres in this manuscript appear to refer to the BNF quoted range of 0.4–1.0 mmol/L [15–17]. A lack of relevant, well-designed studies in determining the optimal concentration has been noted [5]. Several reviews quoted by Nolen et al. [5] suggest the minimum effective serum lithium concentrations may be as low as 0.4 mmol/L. In the UK, NICE guidance published in 2018 [2], recommends clinicians consider maintaining serum lithium level at a relatively conservative range of 0.6–0.80 mmol/L, or 0.8–1.0 mmol/L in people who have relapsed whilst taking lithium, or people who have sub-threshold symptoms with functional impairment. More recently, Nolen et al. [5], as part of the ISBD/IGSLI Task Force on treatment with lithium, concluded that serum lithium concentration should be maintained at 0.6–0.8 mmol/L, with the option to reduce to 0.4–0.6 mmol/L in cases of good response but poor tolerance; or an increased concentration of 0.8–1.0 mmol/L in cases of insufficient response but good tolerance. A controlled study by Gelenberg et al. [18] found that patients randomly assigned to a “low” lithium level (0.4–0.6 mmol/L) had fewer side effects but more illness episodes than patients in the “standard” lithium group (0.8–1.0 mmol/L). However, the lithium levels of some of the patients in the low-lithium group decreased relatively rapidly from their previous treatment levels, a decrease that could have increased their risk of relapse. It must be noted that lithium monitoring is an individualised process, and clinical team must be confident to tailor dosages as best suits the person taking lithium. A number of individuals in our cohorts may be achieving therapeutic benefit at a lower serum lithium concentration, and the prescribing clinician may have chosen to maintain this, rather than risk additional side effects with an increased dose. This may therefore be reflected in both our findings and those of Nikolova et al. [4], who also identified a large proportion (42.4%) of cases with levels below the recommended 0.6–1.0 mmol/L.

Those patients with lower blood lithium concentrations (< 0.4 mmol/L) comprised 19.2% of cases overall. This is higher than that described by Parton et al. [19] who identified that, in a study of 2776 patients with affective disorders from 35 UK MHUs, lithium levels were below 0.4 mmol/L in approximately 10% of patients. This difference is unlikely to be due to the source of the requests as out equivalent data for MHUs was similar to the overall figure at 21.1%. Those with undetectable levels may reflect lack of adherence to medication, while those with low but detectable levels (0.1–0.39 mmol/L) may indicate partial adherence or other scenarios such as up-titration of lithium following initiation of treatment or monitoring after a phase of lithium toxicity. Whilst the majority of these appear to be managed in GPs or MHUs, a larger proportion of these tests were requested in acute or secondary care than those with results within the therapeutic range.

Approximately 5% of results could be defined as over-treated (range 1–1.39 mmol/L). However, this may reflect people who have not yet stabilised their dosage or, for those requested in acute or secondary care, monitoring those experiencing toxicity-associated symptoms. In addition, this group of results may include people who have had samples taken less than 12 h post previous dose. These proportions are again in keeping with the findings of Nikolova et al. [4], who identified levels above 1 mmol/L in 6.9% of cases. Reassuringly, only a small proportion of results (1%) were within the toxic range (> 1.4 mmol/L), and a large proportion of these results were requested by either in acute (38.0%) or secondary (25.9%) care, suggesting an appropriate response to potential toxic side-effects.

### Requesting intervals

Examining the overall patterns of testing frequency (Fig. 2a); we noted that there were multiple spikes of requesting it weekly intervals. This would indicate a tendency for attendance and phlebotomy at clinics on the same day each week within GP practices and MHUs. This has been seen elsewhere where regular testing is required, both by us [9] and others [20].

According to clinical guidance, monitoring serum lithium concentration at regular intervals is necessary, depending on individual status. More frequent monitoring is recommended for those beginning or changing lithium dosage, changing other medications or experiencing intercurrent illness (1 week intervals); and less frequent monitoring is recommended for people who are stable (3–6 months) [2]. Given this advice, it might be expected that frequency plots would show three major peaks, corresponding to populations of unstable therapy (1 week) and at stable therapy (at 3 and 6 months), with a further peak at 1 day for those with lithium levels in the toxic range. Our data indicates that this is broadly true. However, there was a large number of tests performed at non-recommended intervals that are outwith guidance, and there was no evidence of any defined peak at 6 months. In some cases, these tests will be appropriate: for example, people unable to attend their 3-monthly appointment may attend one shortly before or after; or those who become unwell.

NICE guidelines recommend maintaining serum lithium concentration between 0.6 and 0.8 mmol/L for most people taking lithium, with a higher concentration of 0.8 to 1.0 mmol/L for individuals who have had previous relapse [2]. In the absence of other factors affecting lithium, these patients could be expected to adhere to a 3-monthly monitoring regimen. Although it can be seen that the peak representing the most common interval until next test for these results was around 12 weeks, with a smaller peak at 7 days, it is clear that the majority of results are not being repeated within an appropriate time frame; either too early or too late. Further analysis shows that, for those with these lithium concentrations of 0.4–0.99 mmol/L, 36.7% of tests were requested before 11 weeks, 22.5% between 14 and 22 weeks and 10.2% after 27 weeks.

The absence of a significant peak of testing at 6 months likely relates to the logistics of testing; most lithium clinics in the UK are configured to test at 3-month intervals and local shared care agreements for the centres covered made no mention of 6-monthly lithium monitoring [15–17]. A significant number of tests (22,732) were performed in those aged < 65 years of age whose lithium concentration was in the range 0.4–0.99 mmol/L, where 6-monthly lithium testing is indicated, so we would have expected to see a clear peak at this time point if NICE recommendations were being followed. This was not evident on the frequency plots, though we did identify that those aged < 65 years were more likely to have longer retest intervals. Interestingly, Collins et al. showed that age < 65 years was linked to reduced likelihood of following NICE audit standards for lithium monitoring [12], suggesting a reason for the lack of a noticeable peak at 6 months. Following the guidance regarding 6-monthly testing would save up to 6644 lithium tests per year, which, if extrapolated to a UK population would equate to around 200,000 fewer tests per year (equivalent to approximately £250,000 per year). Clearly, a number of these patients will have more frequent tests for other reasons, though it does appear that the 6-monthly guidance is largely not being followed, leading to excessive inappropriate testing.

Conversely, there some people for whom the interval between tests was more than 12 months, perhaps indicating challenges with attendance in this patient group [21].

We did identify a significant association between sex and retesting interval with women demonstrating generally shorter intervals. This is not expected from guidance which does not discriminate between males and females [2, 6], while Collins et al. did not identify any significant associations with sex [12]. However, our large sample size is powered to detect differences that are not clinically meaningful and the observation of an odds ratio of only 1.15 suggests that this is not clinically important.

Reassuringly, for results outside the NICE and BNF recommended lithium concentrations, the repeat intervals were generally shorter. The toxic limit for lithium is usually taken as > 1.4 mmol/L, and for results at this level and above, the majority (60.5%) were repeated either same day or next day and over 87% within 7 days. However, a significant minority (12.9%) were repeated more than 1 week later. As discussed previously, results at this level are usually managed in acute or secondary care, and likely represent active monitoring of lithium overdose. Those requests with lithium levels in the range 1.0–1.39 mmol/L also showed a shorter re-testing frequency, but with a generally longer interval than those with toxic levels. However, again, there were a significant number that were not re-checked within 1 week (*n*=1462; 64.6% of requests). Overall, these may represent those with previously toxic levels under closer monitoring, or those patients who are more disengaged from the service.

### Strengths and limitations

Our study utilises data on large numbers of patients across three UK sites, and highlights the potential of laboratory-based data to examine longitudinal monitoring in a range of conditions [9–11]. However, this route of data collection has the disadvantage of not allowing collection of other clinical data such as diagnosis, time of treatment initiation, lithium dosage, etc., which may assist in the interpretation of the data. The three sites reflected differing models of management of people with bipolar disorder, with each representing mixed urban and rural populations. For example, monitoring of people on lithium therapy served by the UHNM laboratory is predominantly managed by MHUs, the majority of lithium requests for patients served by the PAT laboratory were arranged in primary care, while those served by SRFT were approximately equally distributed between primary and specialist mental health care. This facilitated assessment of the generalisability of our findings.

Compared with some studies [12, 22], we were not able to determine from clinical laboratory records specific information on the reason for each lithium test request or the underlying primary psychiatric diagnosis. In addition to its use in the treatment and prophylaxis of bipolar disorder, lithium is licenced in the treatment of mania, treatment-refractory recurrent depression and aggressive or self-harming behaviour [6, 12]. In an excellent review of conformity to lithium monitoring across 38 UK mental health trusts by Collins et al. [12], the authors showed that around 60% of patients on lithium therapy had a primary diagnosis of bipolar disorder, 25% had unipolar depression and 11% had a psychotic spectrum disorder. While we recognise that this is a potentially important limitation, the recommendations for lithium monitoring within national and international guidance are consistent regardless of indication [2, 23, 24], and many Local Shared Care Agreements focus on lithium therapy, rather than specific primary diagnosis, as a focus for monitoring [15–17]. We do accept that the lack of this information did not allow us to stratify findings by primary diagnosis, and this represents an ongoing challenge in the use of the otherwise extremely valuable laboratory-based datasets.

Our data is also based on the presence of at least one lithium test and may therefore underestimate those who are on lithium treatment, but who are not tested. However, our data does agree with those of other studies in terms of tests per year. However, in addition, our study examines each result and its follow-up interval on a patient-by-patient basis, thereby giving a more detailed view of intervals between requests. Furthermore, our data is based on a large number of patients and is consistent across three sites over 6 years with differing models of distribution of care between general practice and specialist mental health care.

## Conclusions

In summary, our findings indicate that; (a) there is a tendency to manage patients at levels at the lower end of the NICE-recommended therapeutic range, (b) those with elevated levels are frequently managed in acute or secondary care, (c) patterns of lithium results and testing frequency are comparable across three UK sites with differing models of care, (d) intervals between tests demonstrate a noticeable peak at the recommended 3-monthly interval, but there was no evidence of any noticeable peak of testing at 6-monthly intervals, (e) a very large proportion of patients are being monitored outside the recommended intervals and (f) a significant minority with toxic levels are not being monitored adequately. These observations support the need for a review of the recommendations regarding the therapeutic window for lithium and indicate that more needs to be done to improve adherence to the associated guidance on long-term monitoring of lithium levels.

## Data Availability

The datasets used and/or analysed during the current study are available from the corresponding author on reasonable request.

## Abbreviations

NICE: The National Institute for Health and Care Excellence
BNF: British National Formulary
UHNM: University Hospitals of North Midlands
SRFT: Salford Royal Foundation Trust
PAT: Pennine Acute Hospitals NHS Trust
GP: General Practice
MHU: Mental Health Unit
